# Association Between the P2RY12 Receptor Gene Polymorphism and Aspirin Resistance in Patients with Coronary Artery Disease

**DOI:** 10.5195/cajgh.2014.160

**Published:** 2014-12-12

**Authors:** Ludmila Karazhanova, Sholpan Zhukusheva, Ainur Akilzhanova

**Affiliations:** 1 Department of Internal Medicine, Semey State Medical University, Astana, Kazakhstan; 2 Center for Life Sciences, Nazarbayev University, Astana, Kazakhstan

**Keywords:** coronary artery disease, aspirin resistance, gene polymorphism

## Abstract

**Introduction:**

Platelet activation and aggregation are key elements in the development of coronary atherosclerosis. Recent studies have shown that the two polymorphisms of platelet ADP receptor P2RY12 (haplotypes H2 and 34T) are associated with increased platelet aggregation and atherothrombotic risk. It was shown that these polymorphisms promote reduced body response to antiplatelet therapy.

**Aim:**

We investigated the association of P2RY12 gene polymorphisms with aspirin resistance in patients with coronary artery disease (CAD).

**Methods:**

This case-control study included 100 cases with CAD (mean age 57.6 ± 2.8 years) treated in the cardiology department of the city hospital Semey, Kazakhstan, 90 of whom suffered from myocardial infarction. The control group (n = 100) were healthy people without a history of CAD, matched on sex and age. Genotyping of polymorphisms H1/H2 in P2RY12 gene was performed by PCR. Statistical analysis was performed using SPSS v.19.0.

**Results:**

The distribution of H1/H2 genotypes P2RY12 was 42%, 34%, and 24%, respectively, in cases and 42%, 58%, and 0%, respectively, in controls. All allele frequencies were consistent with the Hardy Weinberg equilibrium (*p* = 0.0036 and *p* = 0.0001 in cases and controls, respectively). Genotype H2 was associated with risk of CAD with aspirin resistance (co-dominant model: OR = 3.75, 95% CI 0.14 – 99.88, *p* = 0.05 and dominant model: OR = 2.78, 95% CI 0.11 – 70.93, *p* = 0.05). We found significant differences in the distribution of the mutant genotype H2 between CAD patients with aspirin resistance and healthy controls (χ2 = 30.3, *p* < 0.05).

**Conclusion:**

We found an association of H2 haplotype in P2RY12 gene with aspirin resistance in patients with CAD. However, in order to obtain definitive conclusions about the role of genetic variants with the development of aspirin resistance in patients with CAD, there is a need for further research with a larger sample size as well as the use of selective thromboxane receptor antagonists for studying functional effects of genetic variants.

